# T cells fail to develop in the human skin-cell explants system; an inconvenient truth

**DOI:** 10.1186/1471-2172-12-17

**Published:** 2011-02-18

**Authors:** Bob Meek, Catharina HMJ Van Elssen, Mirelle JAJ Huijskens, Sjoukje JC van der Stegen, Siebe Tonnaer, Stijn BJ Lumeij, Joris Vanderlocht, Mark A Kirkland, Reinout Hesselink, Wilfred TV Germeraad, Gerard MJ Bos

**Affiliations:** 1 Department of Internal Medicine, Division of Hematology, Maastricht University Medical Center+, Maastricht, the Netherlands; 2 PharmaCell BV, BioPartner Building, Maastricht, the Netherlands; 3 Department of Clinical and Biomedical Sciences, Geelong, University of Melbourne, Australia

## Abstract

**Background:**

Haplo-identical hematopoietic stem cell (HSC) transplantation is very successful in eradicating haematological tumours, but the long post-transplant T-lymphopenic phase is responsible for high morbidity and mortality rates. Clark *et al. *have described a skin-explant system capable of producing host-tolerant donor-HSC derived T-cells. Because this T-cell production platform has the potential to replenish the T-cell levels following transplantation, we set out to validate the skin-explant system.

**Results:**

Following the published procedures, while using the same commercial components, it was impossible to reproduce the skin-explant conditions required for HSC differentiation towards mature T-cells. The keratinocyte maturation procedure resulted in fragile cells with minimum expression of delta-like ligand (DLL). In most experiments the generated cells failed to adhere to carriers or were quickly outcompeted by fibroblasts. Consequently it was not possible to reproduce cell-culture conditions required for HSC differentiation into functional T-cells. Using cell-lines over-expressing DLL, we showed that the antibodies used by Clark *et al. *were unable to detect native DLL, but instead stained 7AAD^+ ^cells. Therefore, it is unlikely that the observed T-lineage commitment from HSC is mediated by DLL expressed on keratinocytes. In addition, we did confirm expression of the Notch-ligand Jagged-1 by keratinocytes.

**Conclusions:**

Currently, and unfortunately, it remains difficult to explain the development or growth of T-cells described by Clark *et al.*, but for the fate of patients suffering from lymphopenia it is essential to both reproduce and understand how these co-cultures really "work". Fortunately, alternative procedures to speed-up T-cell reconstitution are being established and validated and may become available for patients in the near future.

## Background

Lymphopenia results in high mortality and morbidity among cancer patients receiving a hematopoietic stem cell (HSC) transplantation, or suffering from HIV infection [1-5]. Eradication of hematological cancers is very successful using haplo-identical HSC transplantation [6], but many patients succumb to opportunistic infections that are the direct consequence of the lymphopenia, mainly involving the T cell pool [7]; it often takes more than 200 days before (mainly CD4^+^) T cell levels have normalized again. This underlines the need for, and explains the general interest in, methods capable of enhancing T cell reconstitution [8,9].

Two important problems associated with slow recovery of T cell levels involve the thymus: slow thymic reconstitution by blood-borne progenitors and thymic involution [10-14]. Because it is still not possible to control and/or reverse either of these processes, there is an obvious need to establish methods that generate a *de novo *T cell repertoire *in vitro*. However, the development of such systems is hampered because most processes that occur in the thymus are still enigmatic, especially how the thymus is capable to enforce self versus non-self recognition on developing thymocytes [15]. Understanding the process of positive/negative selection, and reproducing the process *in vitro*, would potentially help to reduce lymphopenia, especially in older patients as the thymus involutes with age. In this context, the results on thymus-independent T cell development previously described by Clark *et al. *are remarkable. This method involves a seemingly simple co-culture system consisting of skin keratinocytes and fibroblasts grown on a three-dimensional (3D) tantalum covered scaffold (Statamatrix^®^) that, after 4 weeks co-culture with allogenic HSC, results in a population containing 3-5% T cells tolerant to the skin-donor [16]. Even though T cells were detected, only a limited fraction of the expanded HSC actually became T-lineage committed; many cells differentiated towards Class II^+ ^APC, a convenient aspect since it was suggested to be important for CD4^+ ^T cell development [16]. Additional explanations for the extra-thymic development of T cells were the co-incidental expression of various components known to be important for HSC differentiation and thymus-function, such as Delta-Like Ligand (DLL) [8] and AutoImmune Regulator (AIRE) [17] by keratinocytes and fibroblasts [16], respectively.

Even though direct mechanistic explanations for the extra-thymic development of T cells are lacking, and despite limited numbers, it still remains the only published method potentially capable of generating functional, clinical-grade, mature T cells *ex vivo*. Because of the clinical importance, we made an effort to establish the skin-cell system in our laboratory and to characterize it in more detail. We observed that keratinocytes do express the Notch ligand Jagged1, but we did not find the abundant expression of DLL protein previously reported by Clark *et al. *on keratinocytes. Furthermore, due to the growth characteristics of keratinocytes and fibroblasts in the Statamatrix^®^, we could not reproduce the co-cultures as described by Clark *et al. *[16]. As a result, we never observed any T-lineage differentiation. The various reasons for the unsuccessful reproduction of this method are described in this report.

## Results

The *in vitro *T cell development from donor CD34^+ ^cells as described by Clark *et al. *[16] requires a co-culture of (recipient = patient) keratinocytes and fibroblasts. To confirm expression of determinants reported to be important for *in vitro *differentiation, and establish co-cultures, we prepared keratinocyte and fibroblast cell banks from various donors and different skin sources. Sufficient numbers of early passage stocks were available from 5 donors for all experiments.

### Is DLL expressed by keratinocytes?

Many papers have described the necessity for Notch signaling in T cell determination and differentiation from CD34^+ ^cells (reviewed in [8]). One of the most obvious and logical explanations provided by Clark *et al. *[16] for the generation of T cells was the innate potential of matured keratinocytes to express DLL protein. To detect/confirm DLL expression on intact keratinocytes, we used three different methods.

#### FACS analysis

**Figure 1:**
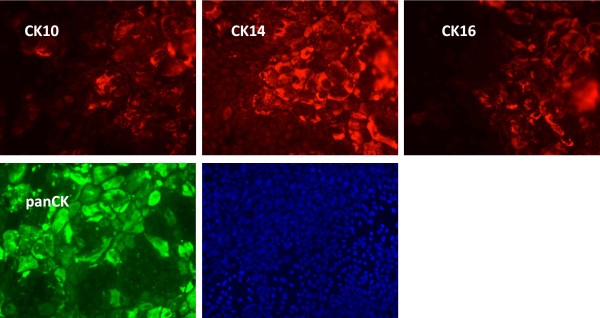
**Differentiation of keratinocytes results in a mixture of immature and semi mature cells**. Keratinocytes of a representative donor were differentiated in chamber-slides for 6 days. Following fixation, cells were stained for the indicated cytokeratins (CK) using a panel of monoclonal (CK10, CK14, CK16) and polyclonal antibodies (pan). Duration of fluorescence detection was kept constant. Though variable, all cells were positive for CK14, while staining of CK10 and CK16 was limited to areas with blast-like keratinocytes.

**Figure 2:**
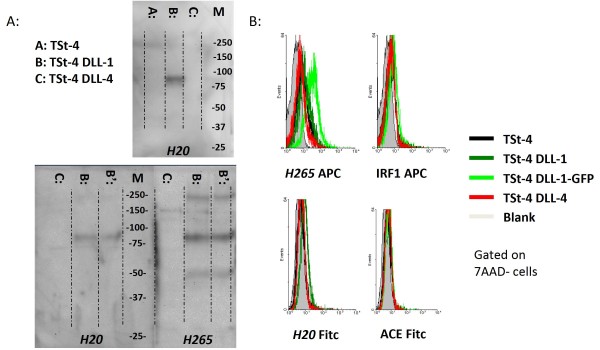
**Analyses of DLL expression by Immunoblot and FACS using cell-lines over-expressing DLL**. (**A**) Protein blots were prepared from TSt-4 thymic stromal cell-lines over-expressing human DLL1 or 4, and incubated with polyclonal antibodies specific for DLL. Both *H20 *and *H265 *only stained lanes containing TSt-4-DLL1 extracts, with a main band at 78 kDa. *H265 *stained additional bands at 50 kDa and 230 kDa, which could represent processed and glycosylated DLL-1, respectively. In contrast to *H20*, *H265 *also heavily stained the marker-lane, indicating that the specificity of this polyclonal antibody preparation is not limited to DLL. (**B**) Indicated cells were harvested using trypsin-EDTA, and incubated with *H265 *and *H20 *+ control polyclonal antibodies specific for IRF1 and ACE. Control antibodies were selected based on species-origin and specificity for non-surface antigens. Both *H265 *and *H20 *showed weak, yet specific staining of TSt4-DLL1.

**Figure 3:**
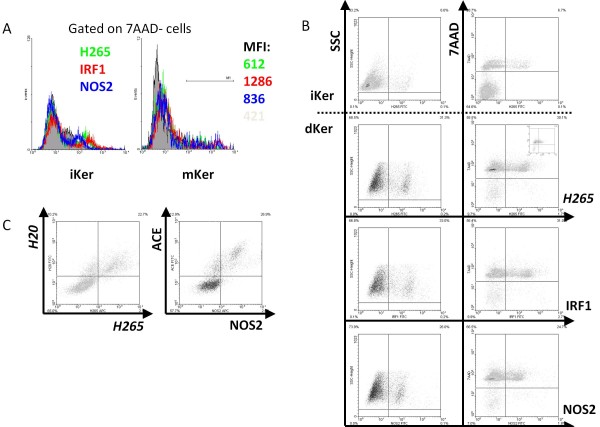
**7AAD^+ ^Keratinocytes show intense staining with any polyclonal primary antibody**. Keratinocytes of donor C were harvested following high-density culture for 6 days, either in standard keratinocyte (iKer) or maturation medium (dKer). (**A**) All cells being stained with indicated rabbit pan antibodies were 7AAD^+^. Left column shows FACS plots as used by Clark et al. [15], right column contains additional information regarding viability. Within experiments, the percentage of positively stained cells was always similar with any pan-antibody. Insert shows background staining by the secondary antibody used. Even though staining intensity was lower after incubation with the secondary antibody, when compared with unstained cells (LL quadrant of the right column), the percentage of cells with signal was similar. This staining-pattern was observed for all donors. (**B**) When gated on 7AAD^- ^cells, any pan antibody stained a low percentage of keratinocytes (donor C, donor D showed similar inconclusive staining profile). (**C**) Almost all cells that were stained with *H265*, co-stained with *H20*. A nearly similar plot could be obtained with any pan rabbit (eg ACE) and pan goat antibody combination (eg, NOS2), and invariably involved 7AAD^+ ^cells (donor C, donor D showed similar co-staining).

In the paper by Clark *et al. *[16], it was not specified at what stage the keratinocytes were harvested, and which DLL was detected by the polyclonal *H265 *antibody. In our hands, the differentiation procedure resulted in a mixture of undifferentiated and early-differentiated cells; none of the "differentiated" keratinocytes ever lost CK14 expression, which is a marker for mature keratinocytes (Figure 1). By using murine cell-lines (genetically) over-expressing human DLL1 or 4 it became clear that the *H265 *antibody used for FACS and IF does recognize DLL1, but not DLL4 (Figure 2A). Using a murine stromal cell line transduced with human DLL1 as positive control, which shows intense staining of DLL1 on immunoblot (Figure 2A), we found that it was possible to detect DLL1 on vital cells by flowcytometry (FACS), albeit with difficulty (Figure 2B). When matured keratinocytes were indirectly stained with *H265 *for analysis by FACS, we were never able to detect a specific signal from *H265 *on vital, 7AAD^- ^keratinocytes (Figure 3A). Other primary rabbit antibodies specific for intracellular antigens, normally not expressed by keratinocytes like NOS2 and IRF1, also stained 7AAD^- ^keratinocytes (Figure 3A), indicating that these antibody-preparations may not be suitable for analysis by FACS. We did find that 7AAD^+^/DAPI^+ ^cells were intensely stained by all primary rabbit antibodies, including *H265 *(Figure 3B), generating a false positive *H265*/SSC picture very similar as published by Clark *et al. *[16]. Apparently, it is possible to use the FACS procedure in combination with *H265 *to detect DLL1 expression on the surface of DLL1-transfected cell-lines, but it cannot be used to reliably stain DLL on vital keratinocytes.

Another question that arose was whether *H265 *is suitable for detection of intact DLL. Because the antibody was generated against a peptide spanning the membrane and the first twenty amino-acids of the extracellular domain, it may recognize processed DLL only (the epitope could be shielded by the 3D folding of the protein), thereby under-representing the actual level of DLL surface-expression. Therefore, using a goat-polyclonal antibody capable of binding to a known, accessible epitope in the extracellular domain of DLL1, *H20*, we repeated the experiments with DLL-transduced cells (Figure 2A and 2B) and keratinocytes (Figure 3C), but no specific staining on intact keratinocytes was detected. When considering the weak specific signals obtained with either *H20 *or *H265 *on cell-lines over-expressing DLL, it is obvious to conclude that it is impossible to demonstrate spontaneous DLL expression on intact primary cells with these antibodies.

#### Immunofluorescence

**Figure 4:**
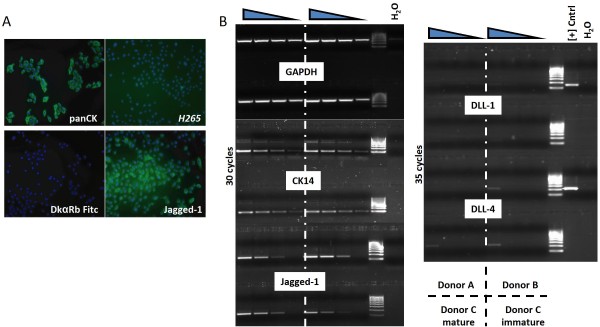
**Keratinocytes have very low expression of DLL**. (**A**): Next to FACS, Clark et al. [15] used immuno-fluorescence to demonstrate DLL expression in immature keratinocyte-cultures. While the secondary antibody did not give any background staining (LL), there were occasional *H265*-FITC positive cells (UR), but these always proved to be DAPI negative. In contrast to DLL, immature keratinocytes were reliably stained for the Notch-ligand Jagged-1. (**B**) For PCR, 4 dilutions (5x-20x-80x-320x) were prepared from cDNA of mKer from 3 donors (A, B, and C), iKer from donor C. Positive controls were prepared from murine TSt-4 cells constitutively expressing DLL-1 or DLL-4. At 35 cycles, DLL4-specific PCR product was only detected in the 5x diluted samples, while mRNA encoding for Jagged-1 was easily amplified in 30 cycles.

To confirm DLL expression by FACS, Clark *et al. *[16] used immunofluorescence on fixed keratinocytes prepared by cytospin, which is an application reported suitable for both *H265 *and *H20*. However, again no detection of any DLL staining of intact, differentiated keratinocytes was observed; the occasional green cell we did see (resembling Figure Five C in [16]) proved to have no nucleus (Figure 4A). In contrast, keratinocytes did stain positive for Notch ligand Jagged-1 (Figure 4A).

#### PCR

Since it was not possible to reliably detect DLL1 or DLL4 protein, we reverted to PCR to find out whether matured keratinocytes are at least capable to express high level of DLL RNA. As shown in Figure 4B, compared with gene-products normally expressed by keratinocytes, cDNA encoding for DLL4 required 35 cycles before detection. Clearly, expression of DLL is far from abundant in differentiated or immature keratinocytes (Figure 4B). In contrast to DLL expression, the high level of Jagged-1 expression was confirmed by detecting PCR products after 30 cycles (Figure 4B). This observation is in line with a previous report demonstrating high levels of Jagged-1 expression at various stages of keratinocyte-development [18].

In summary, using three different methods we could not detect any DLL expression by keratinocytes, which makes it unlikely that HSC differentiation towards T lineage as observed by Clark *et al. *[16] is initiated by T-lineage commitment induced by DLL expressed on keratinocytes. Of course, we do not know whether (or why) keratinocytes start to express DLL during co-culture with fibroblasts, or whether DLL-expression by keratinocytes is actually required for the extra-thymic T cell development. On the other hand, keratinocytes do show abundant expression of Jagged-1. Even though Jagged-1 by itself is not particularly efficient in induction of T-lineage differentiation of cord blood derived CD34^+ ^cells [19], and its expression in the murine thymus is not abundant [20,21], Jagged-1-mediated activation of murine thymic precursors does result in T/NK progenitors [20]. Therefore, it cannot be excluded that Jagged-1 does so as well in the context of the skin explants system.

### A balanced co-culture?

**Figure 5:**
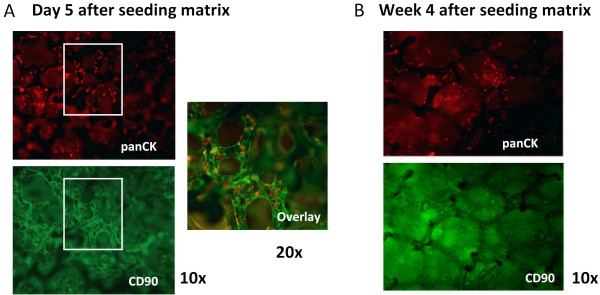
**Keratinocytes are out-competed by fibroblasts**. (**A**) Matrix was stained for keratinocytes and fibroblasts using panCK and CD90, respectively, six days after matrices were seeded at a 2:1 ratio. At this stage, matrices were carefully seeded with HSC. (**B**) After 4 weeks, matrices were completely covered with CD90^+ ^fibroblasts. HSC-derived cells could only be retrieved from the matrix following collagenase-dispase treatment. Fibroblasts from all donors grew similarly.

The system requires co-culture of keratinocytes and fibroblasts on the Statamatrix^®^. However, "matured" keratinocytes showed limited capacity to adhere to collagen-coated Statamatrices^®^, in contrast to fibroblasts, and these latter cells always overgrew in keratinocyte-fibroblast co-cultures in matrices within 7 days (Figure 5A and 5B).

#### Skin explant procedure did not result in T lineage-development

**Figure 6:**
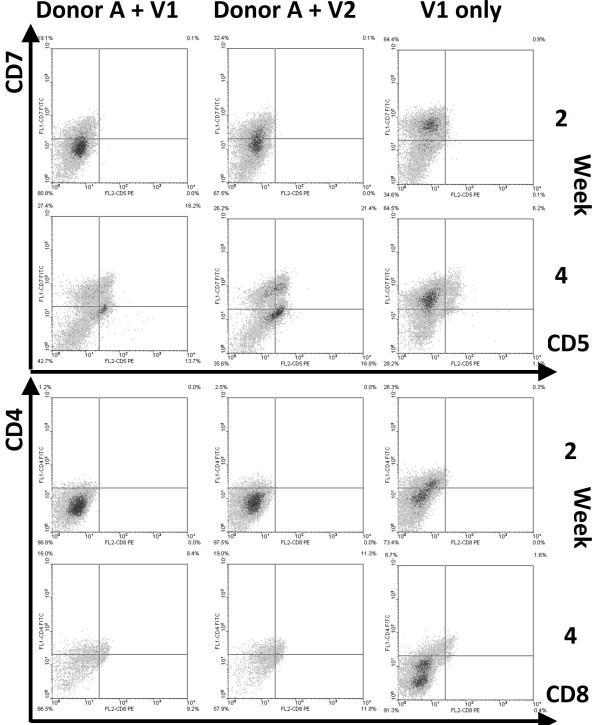
**HSC expand, but do not differentiate in the skin-cell explants**. HSC from donor V1 and V2 were seeded into skin-cell explants from donor A and B. As controls, HSC were expanded for 1 week using a HSC expansion-mix, followed by maintenance with IL-7. For reasons unknown, cell-numbers declined after 2-3 weeks in culture with skin-cell explants (not shown). The phenotype of the hematopoietic cells was analyzed at various time-points after seeding, and the results of week 2 and 4 are depicted. Of markers associated with T-lineage commitment, only CD7 was detected on 20-30%/60% of the cells in skin-cell explants/controls, and its expression-level increased from week 2 to 4.

We found that HSC (expressing either CD34^+ ^or AC133^+^) were never able to differentiate towards T-lineage cells in matrices containing isolated and cultured fibroblasts and keratinocytes; most HSC did up-regulate CD7 and lost the CD34 marker, but no expression of early T-lineage markers like CD5 (Figure 6), nor the CD56 NK cell marker were observed (data not shown). In contrast, the same batches of HSC demonstrated excellent T lineage differentiation capacity when grown on murine stromal cell lines expressing human DLL [22].

#### DLL-independent development?

**Figure 7:**
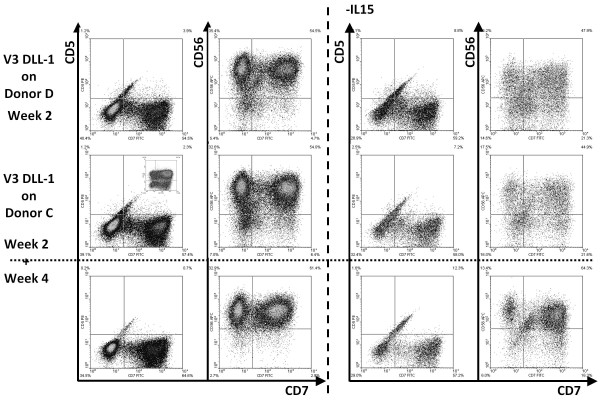
**Skin-cell explants cannot maintain T-lineage committed cells**. HSC from indicated donors were pre-differentiated on monolayers of thymic stromal cells expressing huDLL-1 (see figure 2). After 4 weeks, 55-65% of the cells were CD5^+^CD7^+ ^(see insert UL), and > 90% iCD3^+^CD45RA^+ ^(NS). 1 × 10^5 ^cells were seeded in skin-cell explants and cultured for 2 and 4 weeks. In the presence of IL15, the population expanded 10-15 ×. Already at 2 weeks, all cells had lost expression of CD5 and become CD56^+ ^NK-lineage committed cells. The same phenotype was observed when IL-15 was omitted, although overall expansion was strongly reduced to 1-1,5 ×.

As indicated, the phenotype of the differentiated HSC suggested lack of Notch signaling, which could be due to the lowered keratinocyte/fibroblast ratio. Since it seems very difficult to alter that ratio with the published protocol, it may be difficult to routinely use the skin explants procedure. However, the explants system remains unique in the fact that it encompasses complete T cell development resulting in functional, single-positive CD4 and CD8 T cells, which includes the poorly understood process of positive/negative selection. Because canonical T-cell development roughly consists of 3 phases - 1) DLL-dependent T-lineage commitment, 2) DLL-independent beta-chain selection, and 3) DLL-(in)dependent positive/negative selection, we investigated whether T cell development could occur with cells that already have received DLL-signaling and are T/NK or completely T-lineage committed [22]. Unfortunately, when T/NK-lineage committed cells were seeded in skin-explants, most became NK-lineage committed CD56^+ ^cells (Figure 7), in the presence of additional IL-15. Thymocyte-like cells were never detected. When IL-15 was omitted, the viability and yield of the cultures were reduced considerably, and most cells lost CD5 and iCD3 expression (Figure 7), indicating that even in the presence of IL-7 and Flt3-L, the keratinocyte/fibroblast co-cultures were not able to maintain the T-lineage committed status of cells. The few cells remaining also displayed a CD56^+ ^phenotype.

## Discussion

Our results clearly demonstrate that the keratinocyte differentiation procedure results either in fragile cells that fail to adhere to any structure or plate, or cells that do adhere but are outcompeted by fibroblasts within a week. Furthermore, differentiated keratinocytes do not express detectable levels of DLL (protein nor RNA), and therefore the T-lineage commitment observed by Clark *et al. *[16] must be mediated by other factors. Jagged-1 may be responsible for steering development towards the T-lineage of a limited fraction of CD34^+ ^cells in their co-culture system, because it is abundantly expressed by keratinocytes. As stated in the results section, certain T lineage committed stadia can complete their development into DP and SP T cells independent of Notch signaling [23].

However, even if T cells can be generated *ex vivo *using the skin explants system, the question remains whether they will be functional. Extra-thymic development of T cells has been described for Oncostatin M (OncM) transgenic mice, where LNs take over the function of the thymus [24]. Even though in OncM mice the T cells appear to go through the same developmental stages as regular thymocytes, they are less functional, which may be the result of positive selection by other hematopoietic cells instead of thymic epithelium [25]. This, however, remains to be elucidated for T cells derived in any *in vitro *cell differentiation system.

The past decade knowledge-driven research has revealed how T-lineage commitment can be induced in HSC *in vitro*, using cell-lines transfected with huDLL [8,26,27], or, to a certain extent, with recombinant huDLL [28,29]. In contrast to mouse, differentiation of human progenitors rarely get past the DP-stage of thymocyte development, indicating it is difficult to push them through the positive-negative selection process. Interestingly, experiments with human T-progenitors sorted as or arrested at a DN2-3-like stage, obtained from either OP9-DLL or TSt-4-DLL - HSC co-cultures, respectively, demonstrated that these cells were capable of rapidly reconstituting the thymus of Rag2^-/-^γ_c_^-/- ^mice, where they completed their maturation [22,30]. Similar experiments with murine T-progenitors have shown that this approach considerably enhanced the generation of functional T cells after HSC transplantation [31,32]. This approach will certainly be applicable to patients in the near future, but still relies on the functional capacity of the thymus, which is limited in older patients. In this regard, more pragmatic research has resulted in several viable approaches capable of improving thymic function [33-35], which are now being tested in clinical trials [36,37]. The solutions to both problems concerning T cell reconstitution are expected to meet within the next decade.

## Conclusions

The results described by Clark *et al. *[15] are fascinating and unique, but remain elusive. The results in the current paper show that by following the published procedure we could not obtain enough evidence to support the basic skin cell co-culture conditions suggested to be required for HSC differentiation towards mature T cells. Recent advancements in the field of T cell lineage differentiation and thymic rejuvenation will generate alternative procedures to reconstitute the T cell population following HSC transplantation of man.

## Methods

### Cells

Abdominal or breast-skin was obtained from healthy individuals (donor A-E) undergoing reconstructive surgery, and processed within 24 hours. When required, skin was kept in RPMI 1640 (Sigma-Aldrich Co., St. Louis, MO, USA) supplemented with 100 IU/ml penicillin, 100 μg/ml streptomycin (P/S) and 0,5 μg/ml fungizone (all from Invitrogen Ltd., Paisley, UK) until processing. After removal of subcutaneous fat (if applicable), skin was cut into small fragments and incubated in phosphate buffered saline (PBS, Sigma) containing 2.4 U/mL Dispase II (Roche, Indianapolis, IL, USA) overnight at 4°C. Epidermis and dermis were separated by tweezers.

To isolate keratinocytes, up to 20 epidermal sheets were digested using 2 ml Trypsin/EDTA (Lonza, Verviers, Belgium) for 5 minutes at 37°C. Following quick neutralization by Trypsin Neutralizing solution (TNS, Lonza), suspension was vortexed after which undigested tissue was removed by subsequent filtration through 500 μm (Nedfilter, Almere, The Netherlands) and 70 μm strainers (BD Biosciences, Erembodegem, Belgium). Cells were spun down at 250 g for 10', and resuspended at approximately 2 × 10^5 ^cells/ml in Keratinocyte-SFM medium containing 1% P/S, 0.39 mM CaCl_2_, bovine pituitary extract (BPS) and epidermal growth factor (EGF) according to the manufacturer's descriptions (keratinocyte medium, Invitrogen). Medium was changed after 2-3 days, and islands appeared after 1-2 weeks. Keratinocytes were passed to new flasks when islands contained 30-50 cells; overall density of flasks was kept at 30-40%. For experiments, keratinocytes used were passaged 6x or less.

To isolate fibroblasts, 10-20 dermal fragments were incubated at 37°C for 1 hour in DMEM/F12 (Invitrogen) medium containing 2.5 mg/mL Trypsin, 0.2 U/mL Liberase Bz3, and 0.2 mg/ml DNAse. Following neutralization with DMEM/F12 containing 15% fetal calf serum (FCS, Greiner Bio-one, Solingen, Germany), cell-suspensions were filtrated and spun down as described above. Cells were seeded at 2 × 10^5 ^cells/ml in DMEM/F12 containing P/S, 15% FCS and 10 ng/ml EGF (fibroblast medium). Fibroblast cultures were split when density reached 100%. These cells were never split more than 8x before use in co-cultures.

#### Mobilized CD34^+ ^stem cells

CD34^+ ^cells were isolated from peripheral blood mononuclear cells (PBMC) obtained from healthy volunteers (V1-3) treated with G-CSF (Neupogen, Amgen Inc., Thousand Oaks, CA, USA) at MUMC+ (Maastricht, The Netherlands). Isolation was performed with the Isolex 300i Magnetic cell selection system v2.5 (Baxter oncology, Brussels, Belgium) using the Isolex stem cell reagent kit (Miltenyi Biotec GmbH, Bergisch Gladbach, Germany) according to the manufacturer's instructions. The positive fraction containing > 94% CD34^+ ^cells was frozen at a concentration of 5 × 10^6 ^cells per ml per vial. CD133^+ ^cell preparations were obtained from Lonza.

Skin and CD34^+ ^stem cells were obtained according to protocols approved by, and guidelines stipulated by the local medical ethical committee, and with consent from the donors.

#### Maturation/differentiation of keratinocytes

Keratinocytes at 30-40% density were harvested using Trypsin/EDTA and TNS (Lonza), quantified and seeded at 100% density in a 1:1 (vol/vol) mix of Keratinocyte-SFM and DF-K medium. DF-K medium is a 1:1 (vol/vol) mix of DMEM and Ham's F12 containing P/S, 0.2 ng/ml EGF, 25 μg/ml BPS and 1.5 mM L-Glutamine (Invitrogen). Medium was refreshed daily. This procedure only worked for keratinocytes isolated from breast-skin and not for abdominal skin.

#### The skin cell construct

Sterilized Statamatrix^® ^(Cytomatrix, Australia) were coated by incubation in PBS containing 100 μg/ml rat tail collagen I (Roche) at 37°C, after which the coated matrices were washed twice in PBS, and maintained in PBS until use. Prior to use, matrices were transferred to non-tissue culture treated petridishes (Greiner), and PBS was aspirated. Keratinocytes and fibroblasts were harvested, resuspended in a 1:1 mixture of keratinocyte and fibroblast-medium (ker-fib medium), and quantified. Subsequently, keratinocytes and fibroblasts were combined at 2 × 10^6 ^and 1 × 10^6 ^cells/ml, respectively, and 50-100 μl of this mix was dripped onto a matrix. After 3 hours at 37°C, 5% CO_2_, matrices were moved to 24-well plates and 2 ml ker-fib medium was added. The skin cell constructs were cultured for 6 days and medium was changed every other day.

#### Seeding of CD34^+^/CD133^+ ^cells

After 6 days, medium was replaced by IMDM (Invitrogen) supplemented with 10% FCS (Greiner), 20 ng/ml IL-7 and IL-15, and 100 ng/ml Flt3-L (all R & D systems, Abingdon, UK), then 1 × 10^4 ^CD34^+ ^or CD133^+ ^cells were dripped onto each matrix. Halve medium change was done 3 times weekly.

##### TSt-4

Thymic stromal cell lines TSt-4 and TSt-4 transduced with hDLL1 or hDLL4 were kindly supplied by Dr. H. Kawamoto (RCAI-RIKEN, Yokohama, Japan) and maintained in RPMI 1640 containing 5% fetal bovine serum (FBS), 1% PS, 1 mM sodium pyruvate, 0.1 mM MEM non essential amino acids (NEAA), and 5 × 10^-5 ^M 2-mercaptoethanol (bME)(all from Invitrogen Ltd., Paisley, UK).

### Flowcytometry

All antibodies, materials and equipment were obtained from BD Biosciences unless stated otherwise, and were used according to the manufacturer's instructions. At different time points, cells were analyzed using different combinations of fluorescein isothiocyanate (FITC), R-phycoerithrin (PE), peridinin chlorophyll-a protein (PerCP)-, and allophycocyanin (APC) conjugated monoclonal antibodies (mAbs). The following antibodies (clones) were used: CD1a (HI149), CD3 (UCHT1, SK7), CD4 (SK3), CD5 (UCHT2, L17F12, MEM32 - Immunotools, Friesoythe, Germany), CD7 (M-T701, 7F3 - Sanquin), CD8a (HIT8a, RPA T8), CD14 (M5E2), CD19 (HIB19), CD34 (8G12), CD38 (HB7), CD45 (2D1, HI30), CD45RA (HI100), CD46 (E4.3), CD56 (B159), CD90 (AS02, Dianova, Hamburg, Germany), CD271 (C40-1457), IFN-γ (25723.11), NKG2A (131411, R&D Systems, Minneapolis, MN, USA), NKp46 (9E2), TCRαβ (IP26 - eBioscience), and TCRγδ (B1.1 - eBioscience). Unconjugated goat and rabbit antibodies used were DLL1 (#H20 and #H265), Nitric Oxide Synthase 2 (NOS2), IFN-γ Responsive Factor 1 (IRF1), Angiotensin-converting enzyme (ACE) (all Santa Cruz Biotechnology, Santa Cruz, CA, USA), for which the following conjugates were used according to the manufacturer's instructions: donkey anti-goat FITC and donkey anti-rabbit FITC or APC (all Jackson ImmunoResearch, West Grove, PA, USA). 7-amino-actinomycin D (7AAD) was used to differentiate between viable and dead cells. For intracellular stainings, cells were permeabilized using perm/wash. Cells were analyzed on a FACSCalibur or FACScan with WinMDI (Joe Trotter, http://facs.scripps.edu/) software.

### Sorting

Prior to differentiation, CD34^+ ^cell preparations were depleted of CD38^bright ^cells and contaminating T and NK cells by cell-sorting using conjugated anti-CD3, -CD4, -CD8, -CD38 and -CD56 antibodies. All CD34^+ ^cell preparations had ≤ 0.05% contaminating mature lymphoid cells. Sorting was performed on a FACSAria (BD Biosciences) with FACSDiva software.

### Immunofluorescence

Upon aspiration of media, monolayers or cells were washed with PBS and fixed with cold methanol: acetone (Merck) 1:1 for 10 minutes on ice. Following fixation, preparations were washed 3 times with PBS at RT, with each wash for 4 minutes on an orbital shaker. Blocking was done with for 30' with 1% normal donkey serum (JIR) in PBS, after which preparations were washed twice with PBS. The following primary antibodies were used, each at the proper, predetermined dilution: CD46 (BD Biosciences), CD90 (Dianova), DLL1, Jagged-1 (both Santa Cruz), pan-cytokeratin (Acris, Hiddenhausen, Germany), Keratin-10 (RKSE60), -14 (RCK107) and -16 (LL025)(all MuBio, Maastricht, The Netherlands). Preparations were incubated with primary antibodies for 1 hour at RT in the dark, after which they were washed 5 times with PBS, followed by incubation with secondary antibody for 1 hr at RT in the dark. Appropriate Texas Red or FITC-labeled donkey anti-rabbit anti-goat, or anti-mouse antibodies were obtained from Jackson Immunoresearch. Following another washing procedure, preparations were post-fixed for 15' with 2% paraformaldehyde in PBS. Then, preparations were washed twice, and covered with mounting medium containing 4',6-diamidino-2-phenylindole (Vector Laboratories, Burlingame, CA, USA). Preparations were analyzed using an Axioplan 2 microscope and Axiovision software (Zeiss, Jena, Germany).

### PCR analysis

RNA was extracted from immature and matured keratinocytes using Trizol according to the manufacturer's instructions (Invitrogen). Following quantification and DNAse treatment, cDNA was synthesized using Superscript III according to the manufacturer's instructions (Invitrogen). PCR was carried out in 20 μl reaction volumes containing ≤ 80 ng or 40 ng RNA equivalent from keratinocytes or TSt-4 cells, respectively. All PCR components were used according to the manufacturer's instructions (iTaq, BioRad, Hercules, CA, USA) 100 nM of each primer (Eurogentec, Liege, Belgium). Primers used were (anneal temperature, optimal MgCl_2_): Keratin-14 f-CACCTCTCCTCCTCCCAGTT r-CATCGTGCACATCCATGAC (63°C, 3 mM), DLL-1 f-CGTCGACTCCTTCAGTCTGC r-TTCTGTTGCGAGGTCATCAG (60.5°C, 3 mM), DLL-4 f-TCCAACTGCCCTTCAATTTC r-ACTGCAGATGACCCGGTAAG (57°C, 5 mM), and Jagged-1 f-CGGCCTCTGAAGAACAGAAC r-CCTCAGAGGCTGAGTGTGTG (62°C, 3 mM). All PCR products were validated by TA-cloning and sequencing according to standard procedures.

## Abbreviations

CK: cytokeratins; DLL: Delta-like ligand; HSC: Hematopoietic stem cells

## Authors' contributions

BM; designed and performed experiments, and designed and wrote the paper. CHMJvE; designed and performed experiments, and helped writing the paper. MJAJH, SJCvdS, ST, and SBJL; performed experiments. JV; monitored the project and analyzed the data. RH; organized research at PharmaCell. MK; designed the project. WTVG; designed the project, designed experiments and wrote the paper. GMJB; supervised the project. All authors read and approved the final manuscript.

## A signed response to: T cells fail to develop in the human skin-cell explants system; an inconvenient truth

Rachael A. Clare and Thomas S. Kupper

Department of Dermatology, Brigham and Women's Hospital, 221 Longwood Avenue, Boston, MA, USA

## Response

The authors describe studies attempting to generate human T cells in the cultured skin system we described previously [38]. Their efforts failed to result in a robust population of keratinocytes on the three-dimensional matrix and they failed to observe T cell differentiation. First and foremost, we agree that if there is not a viable robust population of keratinocytes, T cell differentiation will not occur. We have never observed successful T cell generation in this system in without viable and adherent keratinocytes that were at least as abundant as fibroblasts. We believe that the failure of keratinocytes to adhere and grow on the matrices in the authors' hands is primarily a technical issue. Working with keratinocytes can be difficult. These cells need to be passaged at a very low density, under which they maintain a basal cell-like phenotype. Once these cells reach a high enough density or are exposed to other differentiation signals, they can lose their ability adhere and proliferate. As a brief technical note, we have found it is best to plate cells onto the matrices while the keratinocytes are in a basal cell-like phenotype and then allow them to expand and differentiate in situ on the matrix. This produces the desired population of mature keratinocytes that are an absolute requirement for observing T cell differentiation in the system. These are technical aspects we would've been happy to share with the authors.

In the presence of viable keratinocytes, we do observe T cell generation in the system. We absolutely stand by our results, which are genuine. However, work over the last several years has made us less enthusiastic about this system for different reasons. First, the percentage of viable T cells was never higher than 10% of the total output of the matrices, even under the best conditions. This was not enough to for clinical treatment of patients. In order to scale the system up, we attempted to transition to a two-dimensional culture system using fibroblasts and keratinocytes. Under these conditions, the skin cells grew well but T cell precursors only matured to the double positive stage. Despite a great deal of effort, we were unable to generate single positive T cells in the two-dimensional system. Some aspect of the three-dimensional system appears to be required. We transduced human fibroblasts with human Delta-like ligand 1 (DLL-1) in the hopes of eliminating the need for human keratinocytes in this system, but this was also not successful in producing mature, single positive T cells. The Statamatrix three-dimensional environment has actually been shown to support the survival and multipotentcy of human hematopoietic precursor cells, although the mechanism for this remains unclear [39]. Although the need for three-dimensional environment is an interesting biologic question, we were focused on generating T cells that could be used in patients and so left this question for examination by our colleagues working on T cell differentiation as their primary research focus.

Subsequent to the publication of our manuscript, we also developed concerns about the specificity of the H265 antibody. We generated human fibroblasts transduced with human DLL-1 and found that cells expressing DLL-1 by RT-PCR were not reliably detected by flow cytometry with the H265 antibody, in agreement with findings reported here by the authors. We discontinued using this antibody as a result. At the time of our initial studies, this was the best DLL-1 antibody available. However, the inadequacy of this commercially available antibody does not change the fact that T cells develop in the system.

We share the authors' frustration that there is no good system to generate naïve autologous T cells for use in our patients after stem cell transplantation. In the presence of a viable and robust population of keratinocytes, the explant system does support T cell differentiation but we find that it supports the development of too few T cells to be useful clinically. After several years spent attempting to scale up the system for more significant T cell production, we chose to move on to more promising avenues of research. Once the signals that support human T cell differentiation are more clearly defined, it may be worth revisiting. However, we stand by our results, which were genuine and reproducible.

One thing the authors and others may find of interest are some recent findings in the field of peripheral tissue immunity. We and other have found that T cells generated during a cutaneous immune response home to the skin and remain there long term as a resident cell population [40-42]. The normal skin surface of an adult individual contains nearly 20 billion antigen-experienced memory T cells, each of which is the product of a previous infectious challenge [43]. Similar resident populations of T cells exist in the gut and the lung [44]. We would argue that these T cells, each of which is specific for a known pathogen encountered in the past, have the potential to be more valuable for restoring immunity in patients after transplantation. Even if we could generate millions of autologous naïve T cells, only a very small fraction of these would be specific for pathogens actually encountered by our patients. A better approach may be to transfer the established memory T cells from the hematopoetic stem cell donor to the recipient. We can expand 500,000 T cells from a 0.4cm2 skin biopsy [45]. These cells are antigen-experienced memory T cells with a substantial proliferative capacity and impressive effector functions [43]. We would argue that transferring these T cells, harvested and expanded from a small skin biopsy taken at the time of stem cell harvest, could much more meaningful way to address the immunosuppression our patients face after stem cell transplantation.

In short, we agree with the authors that in the absence of viable keratinocytes, there is no T cell development in the system. We believe that the failure of keratinocytes to populate the explant system in the authors hands is primarily a technical issue, one we would have been happy to help them address. However, even under the best conditions, the system only produces a small number of T cells. Several years of effort in scaling up the system or adapting it to two-dimensional system have failed, at least in our hands, to generate clinically significant numbers of T cells. Additional information on the process of T cell development in humans is needed before we can generate large number of naïve T cells in culture.

The authors and our team have the common goal of improving immunity in our patients after hematopoetic stem cell transplantation. It is unfortunate that the authors, instead of contacting us for assistance in getting keratinocytes to adhere and proliferate in their system, chose to write a manuscript describing the fact that when our system is inadequately reproduced, the results are not the same. We would argue that this is not surprising. However, the results we reported in our initial manuscript are absolutely genuine. If the authors wish, we would be happy to help them address these technical issues, although we believe that expansion and transfer of antigen experienced tissue resident T cells will prove to be a better approach in the long run to restoring patient immunity.

## References

[B1] Storek J Gooley T Witherspoon RP Sullivan KM Storb R Infectious morbidity in long-term survivors of allogeneic marrow transplantation is associated with low CD4 T cell counts Am J Hematol 1997 54 131 138 10.1002/(SICI)1096-8652(199702)54:2<131::AID-AJH6>3.0.CO;2-Y 9034287

[B2] Mackall CL Stein D Fleisher TA Brown MR Hakim FT Bare CV Leitman SF Read EJ Carter CS Wexler LH Gress RE Prolonged CD4 depletion after sequential autologous peripheral blood progenitor cell infusions in children and young adults Blood 2000 96 754 762 10887145

[B3] Connors M Kovacs JA Krevat S Gea-Banacloche JC Sneller MC Flanigan M Metcalf JA Walker RE Falloon J Baseler M Feuerstein I Masur H Lane HC HIV infection induces changes in CD4+ T-cell phenotype and depletions within the CD4+ T-cell repertoire that are not immediately restored by antiviral or immune-based therapies Nat Med 1997 3 533 540 10.1038/nm0597-533 9142122

[B4] Lum LG The kinetics of immune reconstitution after human marrow transplantation Blood 1987 69 369 380 3542077

[B5] Ochs L Shu XO Miller J Enright H Wagner J Filipovich A Miller W Weisdorf D Late infections after allogeneic bone marrow transplantations: comparison of incidence in related and unrelated donor transplant recipients Blood 1995 86 3979 3986 7579369

[B6] Ruggeri L Capanni M Urbani E Perruccio K Shlomchik WD Tosti A Posati S Rogaia D Frassoni F Aversa F Martelli MF Velardi A Effectiveness of donor natural killer cell alloreactivity in mismatched hematopoietic transplants Science 2002 295 2097 2100 10.1126/science.1068440 11896281

[B7] Storek J Zhao Z Lin E Berger T McSweeney PA Nash RA Akatsuka Y Metcalf MD Lu H Kalina T Reindl M Storb R Hansen JA Sullivan KM Kraft GH Furst DE Maloney DG Recovery from and consequences of severe iatrogenic lymphopenia (induced to treat autoimmune diseases) Clin Immunol 2004 113 285 298 10.1016/j.clim.2004.07.006 15507394 PMC2956741

[B8] Zuniga-Pflucker JC T-cell development made simple Nat Rev Immunol 2004 4 67 72 10.1038/nri1257 14704769

[B9] Aqui NA June CH Post-transplant adoptive T-cell immunotherapy Best Pract Res Clin Haematol 2008 21 503 519 10.1016/j.beha.2008.07.001 18790452 PMC2579794

[B10] Douek DC McFarland RD Keiser PH Gage EA Massey JM Haynes BF Polis MA Haase AT Feinberg MB Sullivan JL Jamieson BD Zack JA Picker LJ Koup RA Changes in thymic function with age and during the treatment of HIV infection Nature 1998 396 690 695 10.1038/25374 9872319

[B11] Haynes BF Markert ML Sempowski GD Patel DD Hale LP The role of the thymus in immune reconstitution in aging, bone marrow transplantation, and HIV-1 infection Annu Rev Immunol 2000 18 529 560 10.1146/annurev.immunol.18.1.529 10837068

[B12] Mackall CL Fleisher TA Brown MR Andrich MP Chen CC Feuerstein IM Horowitz ME Magrath IT Shad AT Steinberg SM Age, thymopoiesis, and CD4+ T-lymphocyte regeneration after intensive chemotherapy N Engl J Med 1995 332 143 149 10.1056/NEJM199501193320303 7800006

[B13] Uchida N Tsukamoto A He D Friera AM Scollay R Weissman IL High doses of purified stem cells cause early hematopoietic recovery in syngeneic and allogeneic hosts J Clin Invest 1998 101 961 966 10.1172/JCI1681 9486965 PMC508646

[B14] Chen BJ Cui X Sempowski GD Domen J Chao NJ Hematopoietic stem cell dose correlates with the speed of immune reconstitution after stem cell transplantation Blood 2004 103 4344 4352 10.1182/blood-2003-07-2534 14976038

[B15] Rodewald HR Thymus organogenesis Annu Rev Immunol 2008 26 355 388 10.1146/annurev.immunol.26.021607.090408 18304000

[B16] Clark RA Yamanaka K Bai M Dowgiert R Kupper TS Human skin cells support thymus-independent T cell development J Clin Invest 2005 115 3239 3249 10.1172/JCI24731 16224538 PMC1253623

[B17] Mathis D Benoist C Aire Annu Rev Immunol 2009 27 287 312 10.1146/annurev.immunol.25.022106.141532 19302042

[B18] Nickoloff BJ Qin JZ Chaturvedi V Denning MF Bonish B Miele L Jagged-1 mediated activation of notch signaling induces complete maturation of human keratinocytes through NF-kappaB and PPARgamma Cell Death Differ 2002 9 842 855 10.1038/sj.cdd.4401036 12107827

[B19] Jaleco AC Neves H Hooijberg E Gameiro P Clode N Haury M Henrique D Parreira L Differential effects of Notch ligands Delta-1 and Jagged-1 in human lymphoid differentiation J Exp Med 2001 194 991 1002 10.1084/jem.194.7.991 11581320 PMC2193482

[B20] Heinzel K Benz C Martins VC Haidl ID Bleul CC Bone marrow-derived hemopoietic precursors commit to the T cell lineage only after arrival in the thymic microenvironment J Immunol 2007 178 858 868 17202347 10.4049/jimmunol.178.2.858

[B21] Felli MP Maroder M Mitsiadis TA Campese AF Bellavia D Vacca A Mann RS Frati L Lendahl U Gulino A Screpanti I Expression pattern of notch1, 2 and 3 and Jagged1 and 2 in lymphoid and stromal thymus components: distinct ligand-receptor interactions in intrathymic T cell development Int Immunol 1999 11 1017 1025 10.1093/intimm/11.7.1017 10383933

[B22] Meek B Cloosen S Borsotti C Van Elssen CH Vanderlocht J Schnijderberg MC van der Poel MW Leewis B Hesselink R Manz MG Katsura Y Kawamoto H Germeraad WT Bos GM In vitro-differentiated T/natural killer-cell progenitors derived from human CD34+ cells mature in the thymus Blood 2010 115 261 264 10.1182/blood-2009-05-223990 19828700

[B23] Taghon T Van de Walle I De SG De SM Leclercq G Vandekerckhove B Plum J Notch signalling is required for proliferation but not for differentiation at a well-defined {beta}-selection checkpoint during human T cell development Blood 2008 10.1182/blood-2008-07-168906 18948571

[B24] Boileau C Houde M Dulude G Clegg CH Perreault C Regulation of extrathymic T cell development and turnover by oncostatin M J Immunol 2000 164 5713 5720 10820248 10.4049/jimmunol.164.11.5713

[B25] Blais ME Brochu S Giroux M Belanger MP Dulude G Sekaly RP Perreault C Why T cells of thymic versus extrathymic origin are functionally different J Immunol 2008 180 2299 2312 18250439 10.4049/jimmunol.180.4.2299

[B26] De Smedt M Hoebeke I Plum J Human bone marrow CD34+ progenitor cells mature to T cells on OP9-DL1 stromal cell line without thymus microenvironment Blood Cells Mol Dis 2004 33 227 232 10.1016/j.bcmd.2004.08.007 15528136

[B27] La Motte-Mohs RN Herer E Zuniga-Pflucker JC Induction of T-cell development from human cord blood hematopoietic stem cells by Delta-like 1 in vitro Blood 2005 105 1431 1439 10.1182/blood-2004-04-1293 15494433

[B28] Dallas M Delaney C Bernstein ID Enhanced T Cell Reconstitution by Cord Blood Progenitors Expanded Ex-Vivo Using the Notch Ligand Delta1 Blood 2008 112 1199 10.1182/blood-2006-08-039842 PMC1852253 17213287

[B29] Delaney C Varnum-Finney B Aoyama K Brashem-Stein C Bernstein ID Dose-dependent effects of the Notch ligand Delta1 on ex vivo differentiation and in vivo marrow repopulating ability of cord blood cells Blood 2005 106 2693 2699 10.1182/blood-2005-03-1131 15976178 PMC1366491

[B30] Awong G Herer E Surh CD Dick JE La Motte-Mohs RN Zuniga-Pflucker JC Characterization in vitro and engraftment potential in vivo of human progenitor T cells generated from hematopoietic stem cells Blood 2009 114 972 982 10.1182/blood-2008-10-187013 19491395

[B31] Zakrzewski JL Kochman AA Lu SX Terwey TH Kim TD Hubbard VM Muriglan SJ Suh D Smith OM Grubin J Patel N Chow A Cabrera-Perez J Radhakrishnan R Diab A Perales MA Rizzuto G Menet E Pamer EG Heller G Zuniga-Pflucker JC Alpdogan O van den Brink MR Adoptive transfer of T-cell precursors enhances T-cell reconstitution after allogeneic hematopoietic stem cell transplantation Nat Med 2006 12 1039 1047 10.1038/nm1463 16936725

[B32] Ikawa T Hirose S Masuda K Kakugawa K Satoh R Shibano-Satoh A Kominami R Katsura Y Kawamoto H An essential developmental checkpoint for production of the T cell lineage Science 2010 329 93 96 10.1126/science.1188995 20595615

[B33] Rossi SW Jeker LT Ueno T Kuse S Keller MP Zuklys S Gudkov AV Takahama Y Krenger W Blazar BR Hollander GA Keratinocyte growth factor (KGF) enhances postnatal T-cell development via enhancements in proliferation and function of thymic epithelial cells Blood 2007 109 3803 3811 10.1182/blood-2006-10-049767 17213286 PMC1874572

[B34] Goldberg GL King CG Nejat RA Suh DY Smith OM Bretz JC Samstein RM Dudakov JA Chidgey AP Chen-Kiang S Boyd RL van den Brink MR Luteinizing hormone-releasing hormone enhances T cell recovery following allogeneic bone marrow transplantation J Immunol 2009 182 5846 5854 10.4049/jimmunol.0801458 19380833 PMC2760441

[B35] Kelly RM Highfill SL Panoskaltsis-Mortari A Taylor PA Boyd RL Hollander GA Blazar BR Keratinocyte growth factor and androgen blockade work in concert to protect against conditioning regimen-induced thymic epithelial damage and enhance T-cell reconstitution after murine bone marrow transplantation Blood 2008 111 5734 5744 10.1182/blood-2008-01-136531 18334670 PMC2424165

[B36] Napolitano LA Schmidt D Gotway MB Ameli N Filbert EL Ng MM Clor JL Epling L Sinclair E Baum PD Li K Killian ML Bacchetti P McCune JM Growth hormone enhances thymic function in HIV-1-infected adults J Clin Invest 2008 118 1085 1098 18292808 10.1172/JCI32830 PMC2248326

[B37] Levine JE Blazar BR DeFor T Ferrara JL Weisdorf DJ Long-term follow-up of a phase I/II randomized, placebo-controlled trial of palifermin to prevent graft-versus-host disease (GVHD) after related donor allogeneic hematopoietic cell transplantation (HCT) Biol Blood Marrow Transplant 2008 14 1017 1021 10.1016/j.bbmt.2008.06.013 18721764 PMC2601713

[B38] Clark RA Yamanaka K Bai M Dowgiert R Kupper TS Human skin cells support thymus-independent T cell development The Journal of clinical investigation 2005 115 3239 3249 10.1172/JCI24731 16224538 PMC1253623

[B39] Bagley J Rosenzweig M Marks DF Pykett MJ Extended culture of multipotent hematopoietic progenitors without cytokine augmentation in a novel three-dimensional device Experimental Hematology 1999 27 496 504 10.1016/S0301-472X(98)00053-8 10089912

[B40] Liu L Zhong Q Tian T Dubin K Athale SK Kupper TS Epidermal injury and infection during poxvirus immunization is crucial for the generation of highly protective T cell-mediated immunity Nature medicine 2010 16 224 227 10.1038/nm.2078 20081864 PMC3070948

[B41] Gebhardt T Wakim LM Eidsmo L Reading PC Heath WR Carbone FR Memory T cells in nonlymphoid tissue that provide enhanced local immunity during infection with herpes simplex virus Nature immunology 2009 10 524 530 10.1038/ni.1718 19305395

[B42] Clark RA Skin resident T cells: the ups and downs of on site immunity The Journal of investigative dermatology 2010 130 362 370 10.1038/jid.2009.247 19675575 PMC2922675

[B43] Clark RA Chong B Mirchandani N Brinster NK Yamanaka K Dowgiert RK Kupper TS The vast majority of CLA+ T cells are resident in normal skin J Immunol 2006 176 4431 4439 16547281 10.4049/jimmunol.176.7.4431

[B44] Woodland DL Kohlmeier JE Migration, maintenance and recall of memory T cells in peripheral tissues Nat Rev Immunol 2009 9 153 161 10.1038/nri2496 19240755

[B45] Clark RA Chong BF Mirchandani N Yamanaka K Murphy GF Dowgiert RK Kupper TS A novel method for the isolation of skin resident T cells from normal and diseased human skin The Journal of investigative dermatology 2006 126 1059 1070 10.1038/sj.jid.5700199 16484986

